# Beyond digital shadows: Digital Twin used for monitoring earthwork operation in large infrastructure projects

**DOI:** 10.1007/s43503-022-00009-5

**Published:** 2022-12-28

**Authors:** Kay Rogage, Elham Mahamedi, Ioannis Brilakis, Mohamad Kassem

**Affiliations:** 1 Northumbria University, Newcastle Upon Tyne, UK; 2 Cambridge University, Cambridge, UK; 3 Newcastle University, Newcastle Upon Tyne, UK

**Keywords:** Machine learning, Digital Twin, Earthwork, Data analytics, Data pipeline

## Abstract

Current research on Digital Twin (DT) is largely focused on the performance of built assets in their operational phases as well as on urban environment. However, Digital Twin has not been given enough attention to construction phases, for which this paper proposes a Digital Twin framework for the construction phase, develops a DT prototype and tests it for the use case of measuring the productivity and monitoring of earthwork operation. The DT framework and its prototype are underpinned by the principles of *versatility*, *scalability*, *usability* and *automation* to enable the DT to fulfil the requirements of large-sized earthwork projects and the dynamic nature of their operation. Cloud computing and dashboard visualisation were deployed to enable automated and repeatable data pipelines and data analytics at scale and to provide insights in near-real time. The testing of the DT prototype in a motorway project in the Northeast of England successfully demonstrated its ability to produce key insights by using the following approaches: (i) To predict equipment utilisation ratios and productivities; (ii) To detect the percentage of time spent on different tasks (i.e., loading, hauling, dumping, returning or idling), the distance travelled by equipment over time and the speed distribution; and (iii) To visualise certain earthwork operations.

## Introduction

Earthwork is one of the most expensive and critical work packages in large infrastructure projects. Its operation is complex and involves the deployment of an extensive range of heavy earthmoving equipment, such as excavators, loaders, bulldozers and dump trucks, which represent one of the most critical resources for the performance of civil engineering projects, because they are responsible for getting sites ready and for building and moving materials across sites. Plants, particularly heavy earthmoving equipment, such as excavators, bulldozers and trucks, account for a significant percentage of project costs of construction projects, ranging from 10% in commercial projects to 50% in major infrastructure projects, including highways, rail lines and energy projects (Sartori et al., 2014). However, such equipment is often associated with project delays (Kassem et al., 2019; Ok & Sinha, 2006) and considered a major contributor to onsite/offsite congestion and air pollution (e.g., such equipment produced up to 7% of London’s total Nitrogen Oxide Pollution emissions) (Giusti et al., 2014).

A key challenge in large earthwork projects is how to accurately report equipment productivity and usage (Kassem et al., 2021). Current approaches to monitoring earthwork equipment’s performance involve such methods as timesheets and on-site observation notes; in some cases, these tasks are complemented with data acquired from on-board telematics systems or captured via drones. However, an analysis of such approaches indicates that they are error-prone and impractical for large earthwork projects that involve mixed-equipment fleets and require data reporting from multiple sources (Kassem et al., 2021).

By virtue of the advancement and ubiquity of artificial intelligence (AI), Internet-of-Things (IoT), cloud computing and visualisation technologies, Digital Twin (DT) has emerged to lead new technology progress in data-centric construction management. As a digital innovation, Digital Twin provides an opportunity to reduce emissions, delays and costs linked to earthwork operation. However, it requires reliable and effective methods to track devices, measure their performance, and monitor the overall operation of earthwork activities (Hong & Lü, 2022; Kim et al., 2012). Via bidirectional links (usually through sensors and actuators), the concept of DT connects the digital representation of a system to its physical counterpart, while using data-centric insights (which may be obtained via data analytics and Artificial Intelligence) to monitor and improve the performance of the physical system (Al-Sehrawy & Kumar, 2020; Douglas et al., 2021). Current research on DT concerning the construction industry is mostly focused on addressing challenges related to the design and operation of built assets (Opoku et al., 2021). However, evidence of improved efficiencies of operation on construction sites due to implementation of DT has attracted attention (Greif et al., 2020), leading to a proposition that a DT-based approach to monitoring site performance may offer opportunities for automating and improving construction site processes better than the existing practice.

To address inefficient use of earthwork equipment and ineffective measurement of their productivity while controlling their operation, a DT solution for earthwork equipment is developed and tested in this study by integrating three core components of DT: Internet-of-Things (IoT) sensing devices, AI-based data analytics, and visual dashboard. This DT solution provides near real-time analytical and visual insights into earthwork equipment’s usage by detecting patterns, so as to improve equipment productivity. Tested on a real construction site consisting of a large motorway project in three selected scenarios, the proposed DT prototype shows how different layers of DT interact, with the visual dashboard providing a centralised view for monitoring earthwork equipment’s productivity and operation.

The remainder of this paper is organised as follows: Sect. 2 gives a structured review and analysis of related researches; Sect. 3 presents the DT framework and its system architecture and prototyping process; Sect. 4 describes a case study for testing the DT solution proposed and analyses the results obtained; Sect. 5 offers the conclusion of this study and certain areas for future exploration.

## Related studies

Various digitalisation-based approaches have been explored by other researchers for improving the management of equipment and monitoring their performance. An extensive summary and characterisation of these researches is described in Table 1, including the criteria used to characterise the existing studies on earthwork equipment, which are derived from a representative definition of DT for earthwork equipment. These criteria are necessary to enable a closed-loop and behavioural DT able to collect data from earthwork equipment, link such data to a virtual model in real time, and make data-centric analyses, so as to help evaluate earthwork programmes’ performance and reveal the impact of such variables as resource levels and planning changes, without disrupting operation. The criteria include:

- The availability of three core DT parts, i.e.: IoT sensing devices used to capture data about equipment; AI or ML used to analyse performance; and the visual dashboard used to showcase the equipment and on-site performance and operation in real or near real time.
- The coverage of three key management functions for earthwork equipment, i.e.: visualisation, activity recognition, and productivity estimation.
- The type of IoT data captured in the forms of images, videos and other sensor data formats.
- The automation for the availability of automated data pipelines supported by cloud computing: it is necessary for producing scalable AI and ML to cope with the level of automation required in large civil engineering projects for large footprints and dynamic natures of earthwork operation. The automation in implementing data pipelines is also necessary for meeting the first requirement of real- or near real-time dashboard.
- The types of data analytics provided, including *descriptive*, *diagnostic*, *predictive*, and *prescriptive*. *Descriptive* analytics addresses questions arising from the past until the present moment. For example: “What has happened?” and “What is happening now?” *Diagnostic* analytics examines data to explain the reasons of underlying events and addresses the question of “Why did it happen?” *Predictive* analytics attempts to provide future trends by answering such questions as “What will happen?” and “Why will it happen?” Finally, *prescriptive* analytics provides foresights to decision makers by informing them of answers to “What should be done?” and “Why should it be done?” This categorisation of data analytics types is a modified version of the theoretical framework provided by Matthews et al. (2022) for utilising smart data. The modified version consists of separating the *diagnostic* elements from *descriptive* analytics to form a distinct cluster. This is justified by the fact that *diagnostic* analytics in DT may require more distinct technological capabilities than those required for *descriptive* analytics, such as the ability of DT to hold and query historical data for various scenarios/requirements of end-users (e.g., to investigate delay/traffic causes over the past week) in a contextualised environment (e.g., 3D environment).

**Table 1:** A summary and characterisation of related studies

	Digital Twin parts			Earthwork equipment management			IoT data			Automated data pipelines	Data analytics capabilities				Application description
	IoT Sensing devices	Visual near real-/real-time dashboard	Machine learning	Visualisation	Activity recognition	Productivity estimation	Images	Videos	Other sensors		Descriptive	Diagnostic	Predictive	Prescriptive	
Ahn et al., (2012)	●				●				●		●				Monitored the excavator operational status by using vibration signal analysis
Akhavian and Behzadan (2012)	●	●			●				●		●				Detected the loading and unloading activities of a loader and a truck with 3-axis magnetic-field sensing and 3-axis tilt
Montaser and Moselhi (2012)	●				●	●			●		●				Used RFID to calculate truck activity time and productivity
Ahn et al., (2013)	●		●		●				●		●				Used accelerometer data and different ML classifiers for recognition of an excavator’s activity
Montaser and Moselhi (2014)	●	●		●	●	●			●	●	●		●		Used GPS data and simulation to forecast the productivity of trucks
Zhong et al., (2014)	●	●							●		●	●			Used Wireless Sensor Network and IoT to detect the real-time status of tower cranes for safety applications
Akhavian and Behzadan (2015)	●		●		●				●		●				Used GPS and sensor data embedded in smartphones and different ML classifiers for recognition of loaders’ activity
Said et al., (2016)	●			●		●			●		●	●	●		Used telematics data for analysing equipment fleets' utilization and predicting equipment's failure probability
Bügler et al., (2017)			●	●	●	●	●	●			●				Combined photogrammetry and video analysis to measure the volume of the excavated soil and recognize the activity of trucks
Zhidchenko et al., (2018)	●										●		●		Predicted real-time mobile cranes' movement by using simulation
Kim et al., (2018)	●		●		●	●			●		●				Used smartphone embedded IMU data and different ML classifiers to recognize excavators’ activity and measure the cycle time
Cheng et al., (2019)	●		●		●	●			●		●				Used audio signals and SVM classifiers to recognize activities and measure cycle time and productivity of different heavy equipment
Kim et al., (2019)			●		●	●		●			●	●			Used deep convolutional network to analyse videos recorded at the entrance and exit of construction sites, to create a site access log of trucks, and then use simulation to estimate excavation productivity
Moi et al., (2020)	●								●		●				Used DT to monitor the condition of boom cranes and determine stresses, strains and loads at crane hot spots
Chen et al., (2020)			●		●	●		●			●				Used videos and Convolutional Neural Network for activity recognition and productivity estimation of excavators
Zhidchenko et al., (2020)	●								●				●		Proposed a simplified model to predict cranes’ movement and trajectory faster than real time and prevent collisions
Li et al., (2021)	●			●		●			●		●	●	●	●	Used GPS data of trucks and weather condition data in a simulation model to predict required truck fleet size, devise operation schedules and recommend operation routes for highway maintenance operations in winter
Wu et al., (2021)			●		●			●			●				Used Unmanned Aerial Vehicle remote sensing to monitor work cycles of excavators
Salem and Moselhi (2021)	●	●	●	●	●	●			●	●	●	●			Used wireless sensors and IoT for collecting data, a cloud-based MySQL for storing the data, Artificial Neural Network for processing data on a web-based platform, and data analytics for detecting different factors that may influence the productivity of operations displayed in a dashboard
Kassem et al., (2021)	●		●			●			●		●				Used telematics data and a Deep Neural Network (DNN) model to estimate the productivity of excavators
This study	●	●	●	●	●	●			●	●	●	●	●	●	

The existing literature is analysed and mapped against these criteria, and it is found that except for one study by Salem and Moselhi (2021), other studies cover only two of the three core parts of DT. Most studies are focused on using IoT data and implementing machine learning to recognise equipment activities (e.g., the states of equipment: active, inactive, idle, etc.), rather than measuring productivity. Some researchers, such as Cheng et al. (2019) and Chen et al. (2020), addressed both activity recognition and productivity measurement, while others, such as Bügler et al. (2017), proposed certain approaches for visualising the performance of equipment with the data collected from equipment and construction sites.

A variety of techniques were used by the existing researches to collect data about equipment, including: smartphone sensors (Akhavian & Behzadan, 2015; Kim et al., 2018), radio frequency identification (RFID) (Montaser & Moselhi, 2012), accelerometer (Ahn et al., 2013), images (Bügler et al., 2017), and videos (Kim et al., 2019). In addition, a combination of several methods was used to analyse the data collected on site. Some researchers (e.g. Kim et al., 2018; Montaser & Moselhi, 2012; Wu et al., 2021) adopted descriptive analytics to describe, illustrate or summarise data in terms of certain performance indicators, including cycle time. Studies by Kim et al. (2019), Zhong et al. (2014) and others have advanced their data analytics into diagnostics analytics, which is meant to explain reasons of underlying events (e.g., “Why did it happen?”). For instance, they found that low levels of equipment management could be related to excessive equipment idle time. Said et al. (2016), Salem and Moselhi (2021), Kassem et al. (2021) and Mahamedi et al., (2021a, 2021b), among others, have performed predictive analytics by incorporating statistical modelling and/or machine learning, so as to predict future outcomes based on historical data. Scant attention has been paid to prescriptive analytics, which can assist in decision-making and offer recommendations on what to do next and how to optimise solutions of actions. Generally, the goal of prescriptive analytics is to yield actionable insights (i.e., foresights) rather than merely to monitor data or predict the future (TIBCO, 2022). A prescriptive approach proposed by Li et al. (2021) consists of a model to recommend the most efficient truck fleet size and operation routes for highway maintenance in winter.

The mapping of the existing studies against the selected criteria and the explicit description of their implementations, as shown in Table 1, provide a representative summary of the state-of-the-art research in this field. It is shown that only one research (by Salem & Moselhi, 2021) has attempted to create a data pipeline to streamline data collecting and processing, and it includes a visualisation dashboard as an essential part of DT to display the performance of physical assets. As their research is the state-of-the-art investigation closest to this study, a granular analysis of it has been performed to reveal the communalities and differences between their research and this study, with some fundamental differences identified: (i) their data acquisition system included some “portable components installed on hauling equipment and fixed data storage and pre-processing gateway (Meshlium^®^) on the excavation site” (Salem & Moselhi, 2021). Such combination of fixed and mobile data collection sources results in a challenging implementation on large civil engineering projects and is difficult to upscale; (ii) the data pipeline adopted by Salem and Moselhi (2021) used MySQL and Matlab, which delivered a weaker solution scalability than the solution proposed in this study based on modern scalable cloud technologies, which enables repeatable data pipelines; and (iii) the solution offered by Salem and Moselhi (2021) did not give a holistic view of the construction process by linking equipment to earthwork programmes, with no site map or 3D BIM created.

In a word, Table 1 shows that the existing studies failed to simultaneously fulfil all or most of the DT requirements described at the start of this section, with only one study covering all the three core parts of DT. Moreover, they were mostly focused on activity recognition and descriptive insights. And only two of the studies have implemented automated data pipelines, but their technological choices (the variety of sensors used, database types, and AI environment) did not support repeatable data pipelines, which are necessary in large civil engineering projects.

In summary, in terms of earthwork productivity measurement and operation monitoring, there is not a comprehensive DT approach and prototype meeting all the three core characteristics, i.e.: (i) it has the three core DT parts: IoT sensing devices, machine learning techniques, and real- or near real-time dashboard; (ii) it has the ability to provide data analytics at descriptive, diagnostic, predictive and prescriptive levels; and (iii) it incorporates a data pipeline to enable both automated data pipelines and data analytics in a timely manner. To bridge the gaps above, this study proposes a DT framework for construction sites, while developing and testing a DT prototype of earthwork equipment that can help measure the productivity of earthwork equipment and monitor the performance of earthwork operation.

For on-going effort of commercialising the solution proposed in this study, some commercial solutions in the industry were analysed, especially those from Original Equipment Manufacturers (OEM), finding that most of them rely on telematics data, and two main telematics-based solutions are developed by MachineMax^1^ and Hitachi^2^. The limitations of on-board telematics were first analysed by Jagushte (2017), and they were subsequently experimented by Kassem et al. (2021). Such limitations include: a lack of consensus among OEM providers on the definitions of key variables; varying reporting frequencies (daily, weekly, etc.), especially when a plant-hiring company is involved alongside earthwork subcontractors and a general contractor; a lack of earthwork management functionalities, including the integration with earthwork programmes/tasks and 3D data within solutions; and limited data analytics capabilities. These limitations have not only impacted the usability of the existing solutions, but also restricted their suitability in deployment for earthwork monitoring involving a mixture of OEM fleets, which is often the case in large projects.

## A Digital Twin framework proposed for construction processes

A DT framework is designed in this study to provide an efficient and accurate approach to measure the utilisation level of earthwork equipment and monitor the productivity and operation of earthwork programmes in large infrastructure projects. Based on data produced from BIM methods, IoT, site maps and programmes, the framework implements data pipelines with cloud technologies and applies machine learning for measuring and monitoring on-site equipment performance. A digital dashboard is used to visualise tasks and resource performance against visual representation of construction sites. The conceptualisation of the framework and the technological choice underpinning its prototype are based on the criteria for characterising and analysing the related studies in the previous section. These criteria are reformulated below as general principles to underpin the proposed DT framework and its prototype:

- Versatility: The ability of DT to accomplish a range of use cases for a multitude of actors (e.g., project manager, site manager, and site supervisor) and resource types (e.g., a fleet with mixed equipment types).
- Scalability: The ability of DT to provide a scalable architecture that enables its extension with further functionalities through flexible data input/import and external linking.
- Usability: The ability of DT to provide insights in an intuitive and understandable manner (e.g., through contextualising earthwork equipment performance in a dashboard and 3D/4D environment).
- Automation: The ability of DT to provide a suitable level of automation for its core functions (i.e., data capture, data transmission, AI, and performance visualisation), so as to meet the need for repeatable deployments at scale in large-size projects.
- Accuracy: The ability of DT to consistently deliver accurate outputs (e.g., estimated utilisation rate, productivity level, location, etc.).

### Overview of the Digital Twin framework proposed

**Fig. 1:**
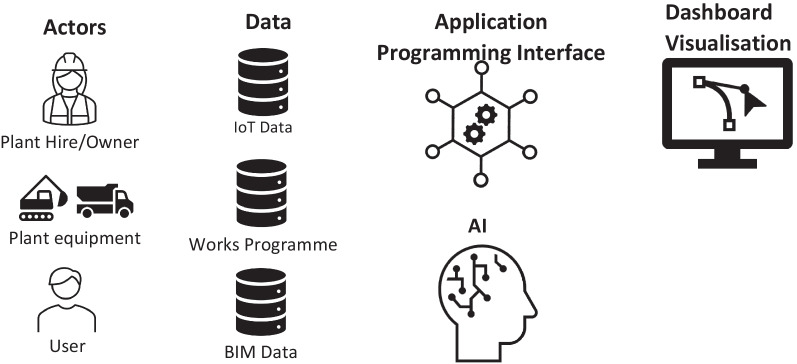
Construction of the Digital Twin framework

The proposed DT framework consists of five key components: (i) system actors; (ii) data; (iii) Application Programming Interface (API); (iv) Artificial Intelligence (AI); and (v) dashboard visualisation (Fig. 1).

#### System actors

The term *Actors* refers to the users and systems that interact with DT. For monitoring earthwork equipment’s productivity, the identified actors include the Plant Hire/Owner, the Earthmoving Equipment, and the User. The Plant Hire/Owner supplies earthmoving equipment, ensures it is maintained and performs as expected, and makes it available when required. The earthmoving equipment itself is an Actor, which interacts with the DT system. IoT data can be captured from the equipment in use. Earthwork equipment is linked to a works programme as a resource, which allocates tasks throughout the construction process. Lastly, the User of the DT system is also an Actor. Whilst the system is primarily designed for Site Manager to acquire an overview of equipment performance in a project, it also provides insights for Project Manager and Site Supervisor. Project Manager is concerned with the overall performance of a site and is responsible for communication with other trades on site and the client. A site manager is responsible for managing the overall site operation, works with several site supervisors, and reports to Project Manager. Site Supervisor is responsible for one or more areas of a site and manages all workface operational measurements, including health and safety, while reporting both to the site manager and the project manager. The research by Kassem et al. (2019) defined the use cases a DT could support for each user role, and those use cases that are addressed in this study are listed in Table 2.

**Table 2:** Digital Twin use cases for user roles

User	Use case
Site manager	1. Equipment usage and productivity 2. Traffic management
Project manager	1. Equipment usage and productivity 2. Traffic management 3. Benchmarking equipment productivity
Site supervisor	1. Equipment usage and productivity 2. Traffic management

#### Data

**Fig. 2:**
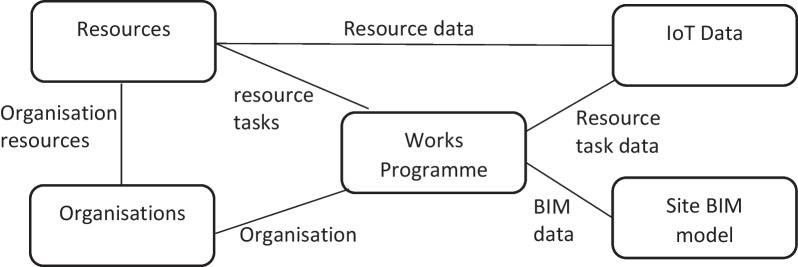
Digital Twin data components

The DT of a construction project requires integrating the data inputted from several static and dynamic data sources. The DT data layer comprises five core data components (Fig. 2). The works programme links together the baseline data for project tasks, schedules dates for task completion, and allocates resources to tasks. Resources refer to physical resources, such as earthwork equipment and personnel. A site BIM model provides both geometric and detailed asset data about a site or the building being constructed. Sensor data track the locations and activities of earthwork equipment. Lastly, data are also required about the organisation delivering the construction project, and such data link the programme to the resource data.

The programme is the central hub to link other data sources together. A construction project consists of a programme, which may have one or more works programmes associated with it. Each works programme consists of a series of time-bound tasks, and each task has one or more resources associated with it. Each resource can be assigned to multiple tasks and can be one of several resource types, including, but not limited to, people and equipment.

In the proposed DT prototype, IoT devices are linked to the equipment assigned to earthmoving tasks, i.e., excavators and dumper trucks. An IMU embedded in a smartphone is used to capture such data as equipment speed, longitude, latitude, altitude, 3-axis accelerometer, gyroscope, equipment identifier, noise level and timestamp from inside the equipment operators’ cabins. A QR code is used to link a resource to an IoT device; thus, IoT devices could be assigned to different resources throughout the project. Sensor data are collected every second and attached to an array of 10 sets of vertices and rows of data BLOBs (a collection of data stored as a single entity is called a Binary Large Object or BLOB). BLOBs are collected every 10 s and posted to an API, which processes the data into a database (hence, the near real-time visualisation of data is achieved). In this way, the data captured can be stored on a phone offline in the event of a connection loss. Once a successful receipt of data is confirmed by the API, the data will be deleted from the device and a new BLOB will then be created. BLOBs are collected in the JSON format and reformatted in the database, so as to make it useable for the system. Data are then linked to a resource object in the programme, which in turn is linked to tasks.

Lastly, the progress data are integrated with BIM data to deliver a near real-time data visualisation of the earthwork operation and the construction progress. Collections of viewable 3D objects within a site BIM model are associated with the tasks within the programme. As these tasks are completed within the programme, the objects can be added to a site view of the project.

#### Application Programming Interface (API)

The API in the DT framework is to link the components together by providing an interface to capture and store data from IoT devices and push the data to the AI engine and the dashboard. In the proposed DT prototype, the API is built based on the OpenAPI specification (The Linux Foundation, 2022). IoT devices post their data to the API, which then registers the data as a JSON string to the database. The JSON data are registered against a resource task within the programme. The dashboard then requests the data from the API by using a combination of API endpoints, with the specific operations depending on the types of information it will visualise from the data. The API specification and endpoints are available at https://aquila.bimacademy.io/api/docs. Via the user interface, users can amend such data as organisation, user, programme, task and resource. Changes to the data are then processed via the API, which would then update the database. Finally, the API interfaces with the AI engine to push CSV data to the engine for processing.

#### Automated data pipeline and AI

Automated data pipeline is a fundamental characteristic of DT, purposed to achieve scalable and repeatable use of DT functions in real-world projects. Data pipelines can process data in multiple formats from heterogeneous data sources with the minimum human intervention, speed up data life-cycle activities, and boost the productivity of data-driven organizations (Munappy et al., 2020). By taking advantage of automation, the implementation of the data pipeline enables traceability, fault-tolerance, and reduction of human errors, so as to create high-quality data (Munappy et al., 2020).

**Fig. 3:**
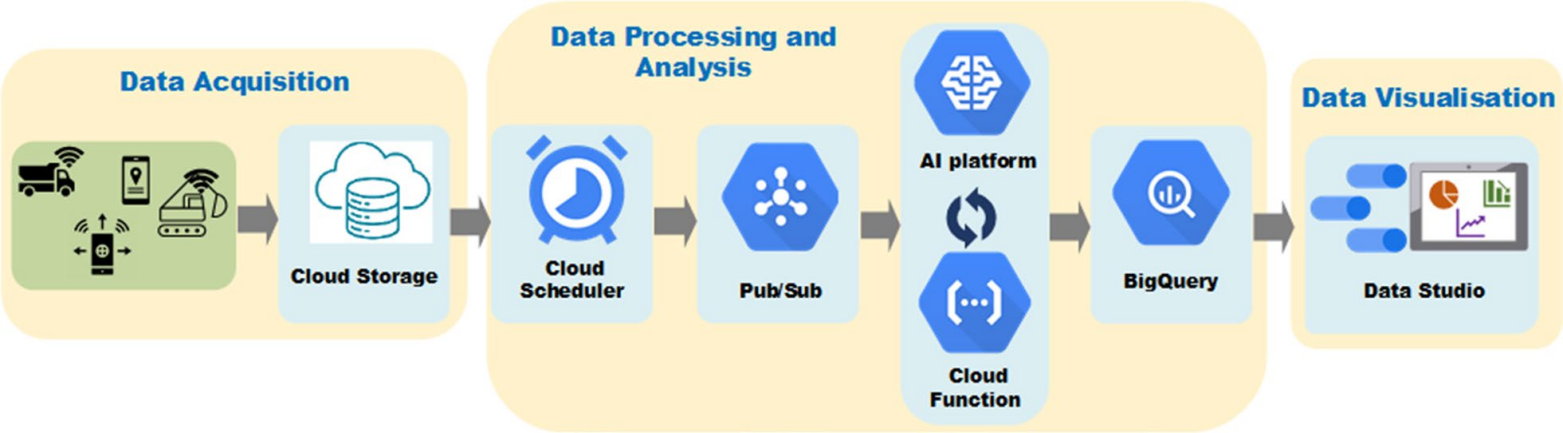
Overview of the automated data pipeline, AI and dashboard

In the DT prototype, a cloud solution is developed to enable automation and repeatability (Fig. 3). As a network-based model, cloud computing provides an easy and on-demand access to computing resources (such as networks, servers, storages, applications and services), which can all be rapidly provisioned and released for use with the minimal management effort and interaction from the service provider (Geewax, 2018). In addition, clouds can offer scalable and affordable computing resources on a pay-as-you-go basis (Bello et al., 2020). The AI engine is also developed on Google Cloud Platform (GCP). Data from the API are uploaded to Google Cloud Storage in the CSV format. Google Cloud Scheduler is set to trigger data processing from storages into BigQuery tables every 5 min. BigQuery is a powerful Google Cloud data warehouse designed to ingest, store, analyse and visualise data quickly and efficiently. As a fully managed data warehouse, Google handles the infrastructure, so that users can concentrate on analysing up to petabyte-scale data (Google, 2021a). The data processing involves running a routine written in Python, which pre-processes the data into a format that can be used by the AI algorithm. The format is required to demonstrate the status of equipment. The status values are generated by comparing values within data rows, so as to detect whether a piece of equipment is in the state of Loading, Hauling, Dumping, Returning or Idling. Once the data are converted, they will be processed with a Deep Learning model on Google AI Platform, and the results will be pushed into the BigQuery tables, which are then queried to create insights from the data by using visualisation, so as to facilitate spotting trends, acting upon them, and making predictions (Google, 2021b). The approach used and the full detail of the deep learning architectures are available from a study by Mahamedi et al. (2022).

#### Dashboard visualisation

**Fig. 4:**
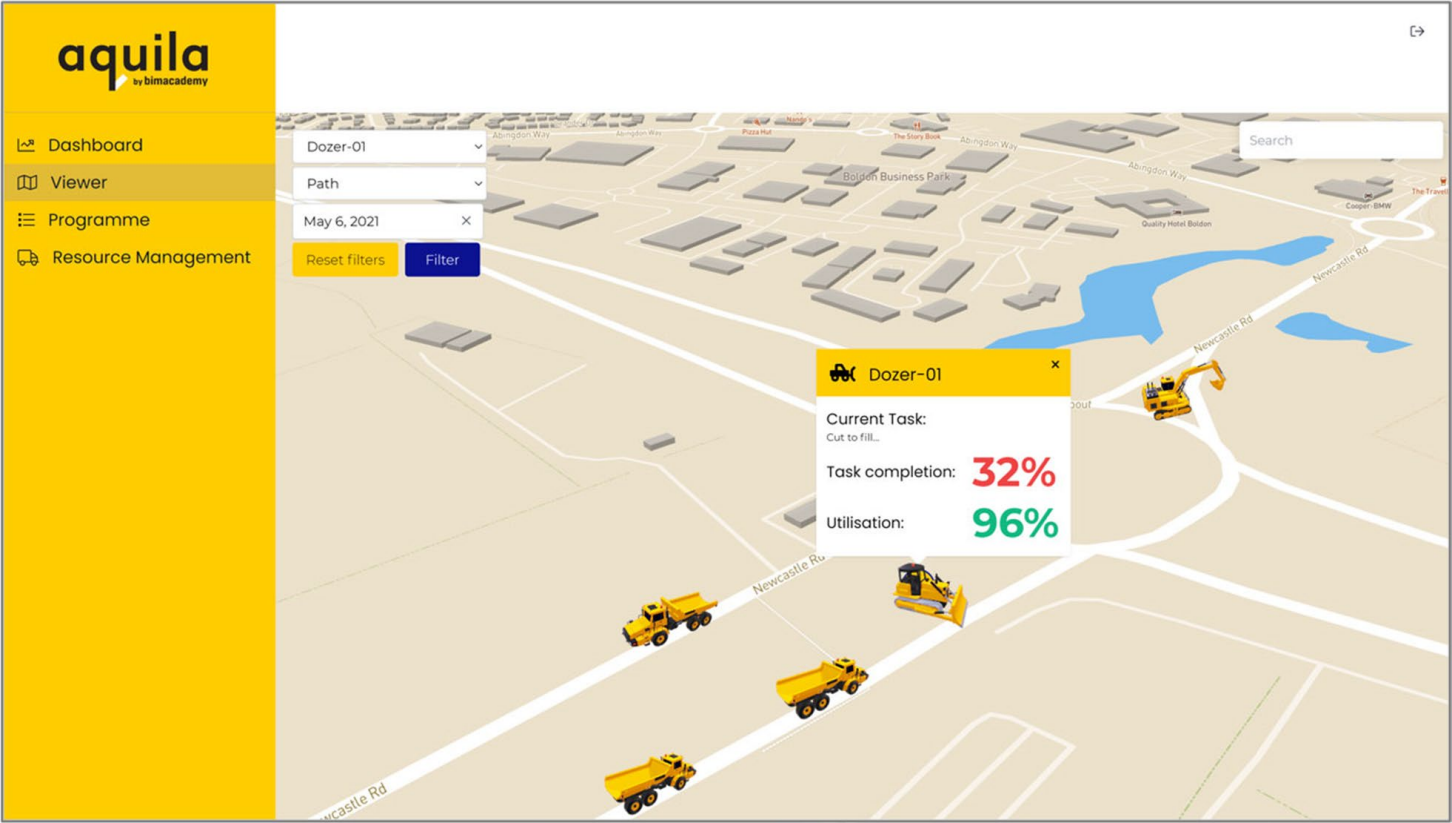
The resource utilisation view

**Fig. 5:**
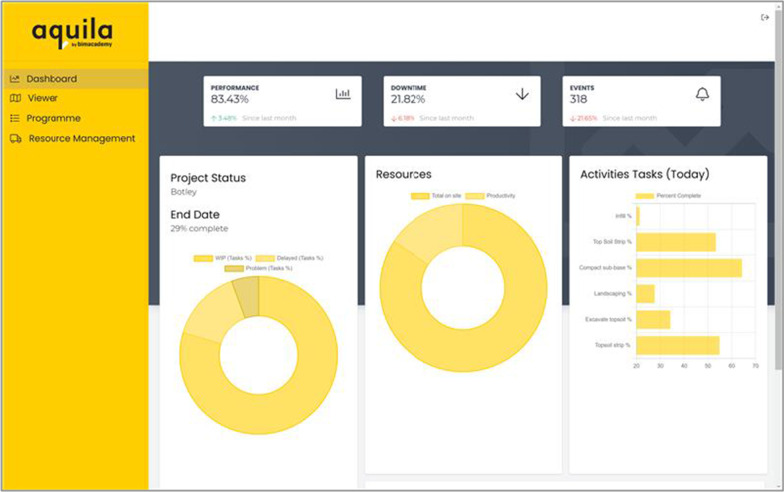
The project performance view

**Fig. 6:**
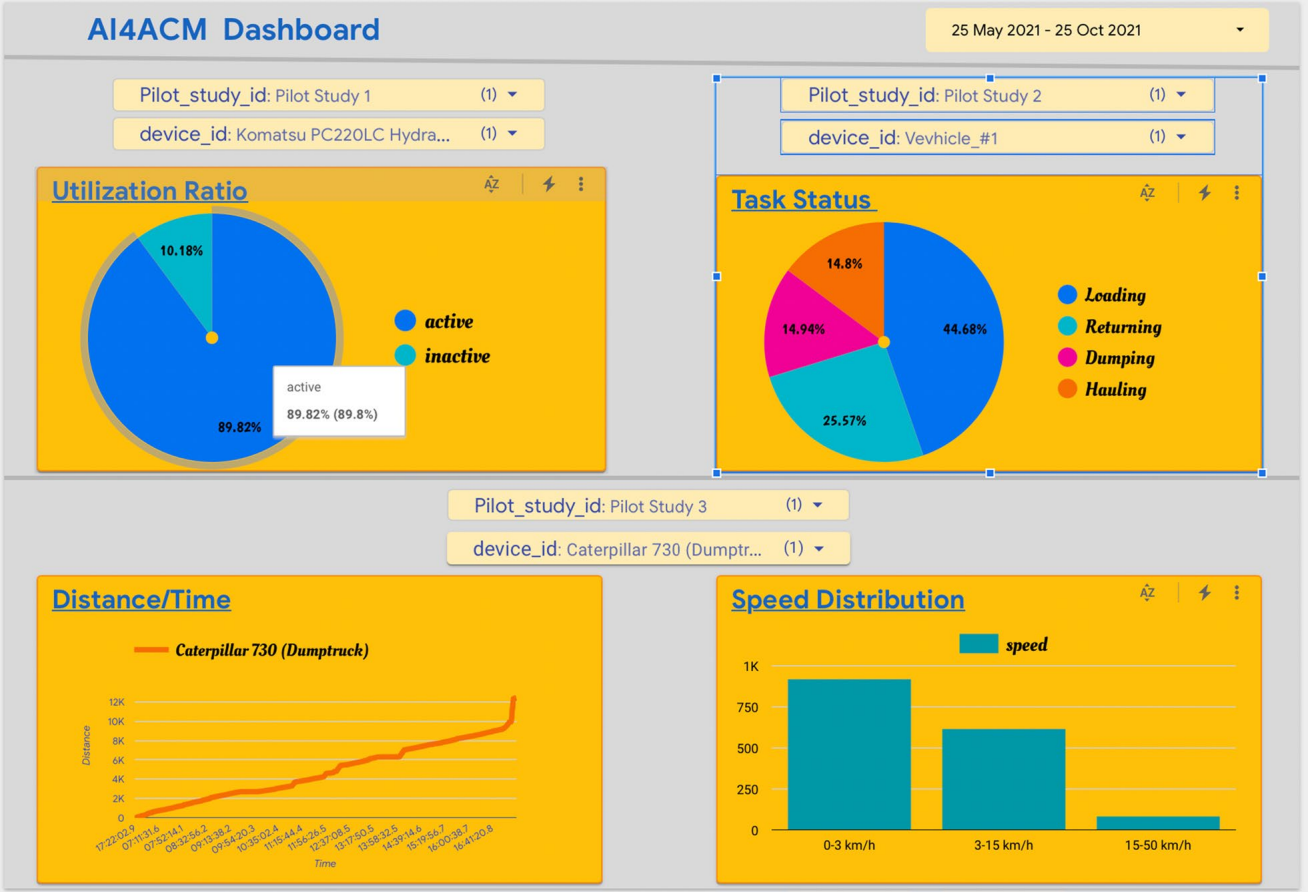
AI engine insights dashboard

Two dashboards are created for this project: Aquila (Figs. 4 and 5) and the “AI for Automated Construction Management” (AI4ACM) dashboard (Fig. 6). Aquila is the commercial name of a development tool for projects (https://aquila.bimacademy.global/), which provides a holistic view of programmes, sites, IoT and AI insight data (Figs. 4 and 5). AI4ACM dashboard (Fig. 6) can visualise some key insights from the AI output data during the development and test processes of AI engines. Aquila is built on Autodesk Forge Viewer, which enables the development of an interactive visual dashboard to be accessed via any web browser. By using the Forge API, Aquila can handle any file formats supported by Forge Viewer; in this way, it can interact with the BIM data used in this study. The dashboard provides an overview of programme progress and resource utilisation. Earthwork equipment resources of a site can be viewed on its geographical representation, which is created by embedding the site BIM model in the MapBox API (Mapbox, 2022) at the geocoordinates available with BIM. As shown in Fig. 5, insights can be visualised on a geographical map for earthwork equipment. And the earthwork equipment objects are animated around the map based on the geolocation data from sensors’ GPS information. Users can observe whether a piece of equipment is moving or whether there are any issues with traffic, e.g., a jam due to breakdown. Users can select a resource to access the task completion and utilisation rates. Task progress can be checked by observing whether resources are provided as planned or whether a mitigation activity is needed. Moreover, users can use the utilisation rate to determine whether a piece of equipment is required for a task.

Sensor data from the monitored earthwork equipment are used to create an overview of the entire project, the resources and the activity performance (Fig. 6).

Finally, machine learning algorithms extract knowledge from the tracked data and provide insights into equipment usages and productivities. Together with the near real-time visualisation of equipment locations and performance, these insights can support Site Supervisors for optimising the project model and improving efficiencies (e.g., to identify lagging performance in a granular way, to pinpoint such cases as congestion, traffic or breakdown, and to take corrective measures, e.g., diverting or removing the existing equipment, or adding new equipment).

An AI4ACM dashboard (Fig. 6) is created within Google Data Studio, which is a free dashboarding tool for establishing connections to various data sources, including BigQuery, so as to provide easy visualisation of data insights. The AI4ACM dashboard is purposed to explore the data and understand what insights could be derived during the AI development process. By using Google Data Studio, quick data visualisations could be delivered without creating specialist tools or knowledge for linking to specialist data, such as programme schedule files or BIM file formats. Four key areas of data insights are revealed from the data: the earthwork equipment utilization ratio, the task status, the equipment distance and time, and the speed distribution. The Utilization Ratio chart shows the percentage of time that a piece of equipment has utilized over the time available. The Task Status indicates the percentage of time that a piece of equipment (in this case, a dumper truck) has spent on different activities within a task. The Distance/Time graph illustrates the distance that a piece of equipment has moved over time. Finally, the Speed Distribution chart demonstrates the speed variation of a piece of equipment in a certain task.

### Systems architecture

**Fig. 7:**
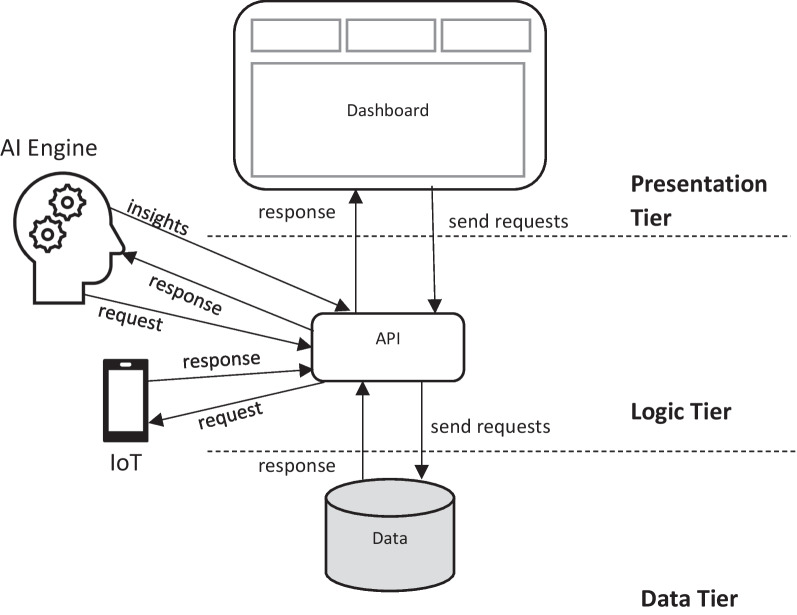
System architecture

The integrated DT currently supports a set of full-stack architecture (Fig. 7), which is composed of three tiers: The presentation tier contains a web front-end viewer; the logic layer is driven by an API for interfaces between the web front-end, a data layer and an AI engine; the data layer coordinates various data sources within a central database. This study uses two separate dashboards to present insights acquired from the AI process. Figures 5 and 6 demonstrate a dashboard developed to link insights from the AI process to the BIM and programme data, for Actors such as the General Contractor, to give them the ability to control the overall project schedule and performance. The AI4ACM dashboard represents the insights acquired from the AI process based on IoT data only. The second dashboard does not consider the works programme or BIM data; it is developed for such Actors as earthwork subcontractors as well as plant and haulage companies; these Actors do not necessarily require an oversight of the whole programme.

The API acts as another engine to capture and process the data from sensors, BIM and programme, so it can be linked and manipulated via the AI engine and dashboard. The API is sensor agnostic and able to consume data from any device that conforms to the API data schema. For example, the case study for testing the DT uses the IoT data (i.e., kinetic, noise and location data) from a smart phone. This pattern provides flexibility in capturing and transmitting data, while helping overcome the challenges present in the current telematics systems, e.g., inconsistencies in the types of data captured, the definition of key variables and their data reporting intervals (Kassem et al., 2021). The database is also decoupled from the API, so it can be maintained as its own entity. Finally, the AI engine has no need to understand how the data are captured and processed; it only requires the input data to conform to the specified schema for processing the data, so as to output the results that the dashboard can visualise. This approach to creating a decoupled DT facilitates to create several customized applications and viewers, so as to present different data insights from the API, as demonstrated in the AI4ACM and Aquila dashboards.

## Case study: deployment and testing

The DT framework and system architecture were implemented as a prototype and validated through a case study in a major infrastructure roadworks construction carried out on the A19 motorway in the Northeast of England. The stakeholders involved within the project included an infrastructure client, a general contractor, a rental and earthworks subcontractor, and several original equipment manufacturers (OEM). The general contractor was responsible for the overall management of the project, covering the project’s planning, co-ordination and delivery. The earthworks company was responsible for providing resource plans, which detailed what resources were allocated to which tasks within the project programme of works. The plant equipment was hired from a rental company, which sourced equipment from different OEMs. The resources used in the project included excavation, shaping and hauling vehicles. Each of these plant equipment categories were manually added to the database, along with the details of each instance of the resources used in the project.

The programme was produced as a digital record created within Primavera P6 Enterprise Project Portfolio Management. And then, the project programme was exported as a Microsoft Excel file from Primavera. The tasks, task duration, task start and finish dates, and the critical path programme data were imported from the Excel file into the API, which stored the data in a database. The resource plan was provided as a hard copy of a manually updated programme of resources. A preformatted form was provided with handwritten notes, which detailed which resources were related to what tasks within the project programme. The resource data were then allocated to the tasks via the API and stored within the database. The IoT devices were installed within the cab of the physical resources and linked to the resources in the API via QR code. This design allowed any IoT device to be assigned to any task. When a device was registered to a new resource, the API would modify the information on which task the device became assigned to, so that devices are equipment agnostic and reusable across different resources. Data were pushed from the devices to the API, which in turn pushed the relevant sensor and programme data to Google Storage for processing by the AI engine. Finally, the output from the AI engine was queried by both Aquila and Google, so as to create insights from the data.

Although a full DT of the A19 motorway project was developed, the limitation measures against Covid-19 prevented site access, so we were unable to capture enough data about the *round trip* of a truck and the AI engine could not be effectively tested on that project. In this study, a *round trip* is defined as the entire process of a truck to load earth, haul the earth to a dumping point, dump the earth, and return to collect more earth, as required under the task assigned to the vehicle. In order to address this limitation, IoT data were captured from another real construction site, so as to demonstrate the AI insights that could be captured from equipment working on a full round trip. Three test scenarios were developed to understand what insights could be gained from the data.

### Three test scenarios

Three test scenarios were developed to demonstrate the following data: earthwork equipment utilization rates; task status; equipment distance and time; and speed distribution of equipment on site.

The first test scenario was to capture the *utilisation ratio* of a Komatsu PC220LC Hydraulic excavator on a demolition project, with a smartphone mounted in the cabin of the excavator. The gyroscope data were captured by a 3-axis accelerometer at the frequency of 8 Hz and then used as the input for training the deep learning model introduced by Mahamedi et al. (2022). The model’s output is the probability of the excavator’s active or inactive status. Video footage of the excavator performing the task was used to compare the predicted task status with the actual one.

The second test scenario captured the *round trip* of a truck transporting earth from one location to another. The round trip covered the loading, hauling, dumping and returning activities of a truck for a task. A smartphone was mounted on the vehicle dashboard, and the vehicle would complete a round trip within a predefined zone, which was split into further areas for each of the activities within a round trip. The loading area referred to the site where an excavator would load a truck, while the tipping area was where a truck would dump soil. A vehicle’s time in the loading and tipping areas was used as its loading and dumping duration, respectively. The time for travelling from the loading area to the tipping area was regarded for hauling, while that from the tipping area to the loading area was regarded for returning. Task durations were recorded manually by vehicle drivers, so as to evaluate the accuracy of the algorithm developed for automatically recognising each task on the round trip.

In the third test scenario, several trucks were working on one earthmoving project. Smartphones were mounted on the vehicle dashboards to capture the time taken by the vehicles to move and the distance for the vehicles to move within that time slot.

### Results

The output from Scenario 1 showed that the PE had a utilization ratio of 89%, with an approximately 99% prediction accuracy of the deep learning model. The details of the Deep Learning models tested with various combinations of data input (i.e., noise, kinetic data, and gyroscope data) can be found in research by Mahamedi et al. (2022). In addition to the benefit of deploying this solution on fleets of mixed equipment with agnostic IoT sensing devices mounted, the results obtained showcased significant improvements in terms of accuracy, compared to the productivity measurements attained with telematics data in research by Kassem et al. (2021). The output of the AI could predict whether the PE was performing as expected. These insights could be used by site and project managers to determine whether the measured equipment performance might impact the overall progress of a project, and to take corrective actions subsequently.

Scenario 2 presented predictions (Table 3) of the percentage of time taken by a truck for an activity within a task. This process involved the delineation of site zones as locations for loading and dumping. When the GPS location of a vehicle was within the known loading/dumping area and the speed of the vehicle was approximately zero, the algorithm would recognize the status of the vehicle as loading/dumping. If the vehicles speed was significantly above zero (i.e., the vehicle was moving), the algorithm would recognise the status of the vehicle as hauling or returning, depending on its previous status. For example, if the previous status was for loading, the current status would be determined as hauling; and if the previous status was for dumping, the current status would be determined as returning. The percentages of actions recognised by the algorithm in a round trip are shown below. The value for each state (44.68%, 14.8%, 14.94%, and 25.57%) is very close to the actual percentage, with a maximum error of 4.3%.

**Table 3:** Prediction of round-trip behaviour

	Loading (%)	Hauling (%)	Dumping (%)	Returning (%)
Recognized behaviour	49	19	15	27
Actual behaviour	44.68	14.8	14.94	25.57
Error	4.3	4.2	0.06	1.43

**Fig. 8:**
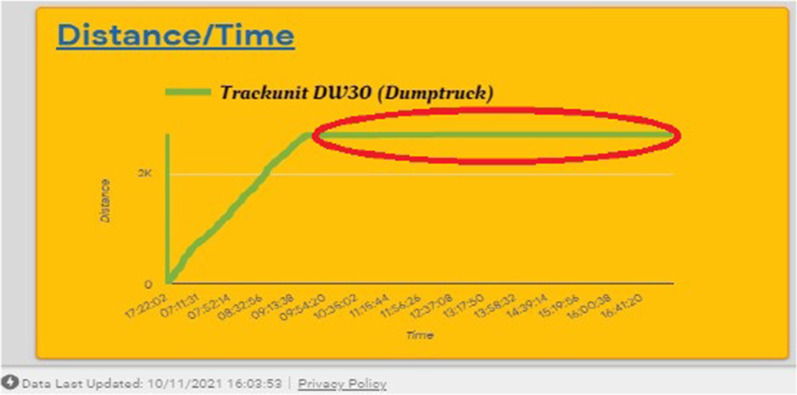
Sample of distribution/time chart with potential bottleneck in a truck operation

The output from Scenario 3 demonstrated the distance for trucks to move over a period (Fig. 8). If there is little movement by a truck compared with the threshold defined by experts, a bottleneck or delay in the truck operation can be decided. An example of such a situation is shown in research by Mahamedi et al. (2022) (Fig. 4). This information can be used by Site Manager to detect any productivity issues. Within this test scenario, it is also possible to analyse the distribution of speeds of equipment. The information about the speed distribution of a truck is useful for planning where a high truck speed is required when estimating the duration of truck activities or the level of truck resources for boosting on-site progress.

## Discussion

Earthwork equipment represents a significant portion of resources in construction projects, while forming a major contributor to onsite and off-site congestion and pollution. It is always on the critical path of most civil and building projects. The existing literature proposes that a more effective approach to measure the productivity and monitor the performance of equipment—can enhance the utilisation of earthwork equipment and the performance of projects (Mahamedi et al., 2021a, 2021b; Salem & Moselhi, 2021). Furthermore, the existing literature suggests that a DT approach to monitoring site performance may offer opportunities to improve earthwork equipment’s utilisation and on-site operation (Kassem et al., 2021). Although some studies have explored how to capture, visualise and process data in AI methods, there is no comprehensive DT approach with the following three characteristics: (i) contain the DT core parts (i.e., IoT sensing devices; real- or near real-time dashboard; and machine learning techniques); (ii) have the ability to provide different levels of data analytics, including descriptive, diagnostic, predictive and prescriptive functions; and (iii) incorporate automated data pipelines to enable on-going/repeatable applications and data analytics in a timely manner.

This paper addresses the identified gap and demand by providing a DT framework for construction sites as well as a DT prototype for measuring the productivity of earthwork equipment and monitoring earthwork operation. The DT framework consists of three layers within a set of interconnected system architecture, which offers different types of data analytics, including descriptive, diagnostic, predictive and prescriptive functions. The DT prototype can capture field data (e.g., data on kinetics, gyroscope, location and speed) from each equipment, apply AI to predict the performance of earthwork equipment by using automated data pipelines and AI in near real time, and present results in two visualisation dashboards (i.e., an Aquila dashboard, and an AI4ACM dashboard). A methodology for a data pipeline that enables automated data collection, pre-processing and analytics in a timely manner based on GCP tools’ functions is also introduced as part of the DT framework and prototype.

The studies reviewed and the commercial applications now available can capture equipment’s operational data either through telematics or IoT sensors, but such data are not linked to earthwork programmes or site 3D models or site maps. This missing linkage limits the usefulness of DT, especially for general contractors who are responsible for the timely execution of earthwork programmes. This defect also limits the ability to derive planning foresights and test various resource levels and their combinations. In the DT framework and prototype proposed in this study, the linking of IoT data from the field to work programmes and 3D BIM and/or geographical site maps was a fundamental element. In this way, site map data and near real-time or real-time updates of earthwork equipment’s locations can provide a holistic overview of sites at any point of time. Furthermore, the application of ML with Deep Neural Networks (DNNs) helps accurately predict earthwork equipment’s productivity in construction works.

This study delivers several advancements over the exiting researches both at the global level (DT solution level) and the local level (DT’s individual components). At the global level, this study makes certain theoretical contributions with a DT framework for construction sites, including some general principles for construction DT. This forms an enhancement to the existing literature in terms of Digital Twin, because the existing literature is mainly focused on the operational or in-use phase of built assets, with very few studies on the construction and production phases of projects. In addition, for practical purpose at the global level, this study presents and demonstrates a full DT prototype for monitoring earthwork equipment’s productivity and on-site operation. The DT prototype proposed is composed of five key components (i.e., system actors, data, API, AI engine, and dashboard visualisation) and instantiates the general DT framework and its principles.

At the local level, this study combines a few advancements in a set of DT system architecture to ensure it is accessible, universal, usable, automated and accurate. In terms of *versatility*, the DT is conceived to accomplish a range of use cases while preserving its ability to operate in a mixed fleet of equipment. Central to this feature is the deployment of smart phones as agnostic IoT sensors, which can be installed across excavators, trucks and other devices of various OEMs. Through the implementation of open APIs, the DT prototype makes DT more *universal* so that it can accommodate further functionalities and link external applications. The DT prototype also enhances *usability* through two dashboards, which help contextualise the operation of earthwork equipment. *Automation* is a key feature in the implementation of cloud-based data pipelines and AI. Finally, the *accuracy* of prediction of earthwork equipment’s productivity and performance is improved compared to using other approaches, such as telematics.

## Conclusions

The insights derived through the DT about the performance of earthwork equipment can directly uncover blind spots in terms of productivity for site managers and project managers, especially in large infrastructure projects. The real implication of this capability to project control resides in the fact that such measurements can be repeated as much as required and implemented in near real time, an advantage that is not possible with the current project control methods. For example, the ability to frequently measure the performance of each equipment on site facilitates the regularity of applying project control techniques (such as Earned Value Analysis), which have been applied less regularly in the current practice, due to the big effort involved in massive measurements on site.

The proposed DT framework and prototype is modular and scalable, thus allowing for targeted improvement and extension of specific elements (e.g., two dashboards are developed for two user groups). However, for effective usage, it requires both quality data and an adequate level of details. For example, the quality of the works programme data, and the details of the resources linked to tasks, and the way the data are captured, are all critical to the success of the proposed DT in construction sites based on the functioning of its AI and dashboard modules.

This study is not without challenges and limitations. One of the key challenges is the Covid-19 restrictions, which prevented the timely access to the case study site and the deployment of IoT devices on a wide range of equipment, causing delays in capturing the data required to develop and optimises the AI engine model. To overcome this issue, an alternative site was used to capture data for the purpose of refining the AI engine and testing the initial solution. When site access was allowed, earthwork equipment operators imposed another barrier to data capturing, because the IoT devices used within the earthwork, which took the form of mobile phones, were often not turned on by the earthwork equipment operators, either because they forgot to do so or refused to do so intentionally, as they might not want their movements to be tracked. This suggests two aspects for consideration in future studies: (i) To examine such operators’ behaviour and concerns when working in digitally twined construction sites, so as to identify their ethical issues; (ii) To develop an IoT technical solution that can autonomously capture data while the earthwork equipment is operating, but without overlooking the aforementioned ethical issues and concerns.

## Data Availability

Additional data will be made available upon request.

## Footnotes

1 https://machinemax.com/pages/solutions.

2 https://www.hitachicm.co.uk/support-service/fleet-management/.
